# Drug Resistance and Cardiovascular Safety of Second-Generation Anti-Androgens in Patients with Advanced Prostate Cancer: Second-Generation Antiandrogen Use in Advanced Prostate Cancer

**DOI:** 10.31661/gmj.v11i.2727

**Published:** 2022-12-31

**Authors:** Venus Shahabi Raberi, Akram Shariati, Mohsen Abbasnezhad, Naser Aslanabadi, Amirreza Abbasnezhad, Amir Bahmani

**Affiliations:** ^1^ Department of Cardiology, School of Medicine, Urmia University of Medical Sciences, Urmia, Iran; ^2^ Cardiovascular Research Center, Tabriz University of Medical Sciences, Tabriz, Iran; ^3^ Faculty of Medicine, Tehran University of Medical Sciences, Tehran, Iran

**Keywords:** Second-Generation Antiandrogens, Efficacy, Drug Resistance, Receptors, Androgen, Cardiosafety, Cardiotoxicity

## Abstract

Prostate cancer is recognized as one of the most common cancers affecting the male population. The prostate is revealed to be a hormone-dependent tissue as testosterone and dihydrotestosterone could bind to the androgen receptor, activate it, and initiate the nuclear translocation of this receptor, followed by subsequent signaling cascades. Regarding this androgen dependency on the prostate, it is believed that androgen deprivation therapies can confront aggressive prostate cancer as a first-line treatment. However, prostate cancer could overcome hormone deprivation strategies through several cellular mechanisms, such as intratumoral androgen production and the production of ligand-independent androgen receptor splice variants, known clinically as castration-resistant prostate cancer. Due to the limited efficacy of first-generation anti-androgens in complete blockage of androgen receptor activity, recently, four second-generation anti-androgens, including abiraterone acetate, enzalutamide, apalutamide, and darolutamide approved by the United States Food and Drug Administration, and considered standard of care for patients with advanced prostate cancer. In addition to some reports of drug resistance treatments, cardiotoxicity, including heart failure, ventricular repolarization, hypertension, myocarditis, atrial fibrillation, and ischemic heart diseases, is commonly observed in patients who underwent abiraterone acetate and/or enzalutamide therapy. However, cardiotoxicity has rarely been observed after treatments of apalutamide and/or darolutamide.

## Introduction

Cancer is considered one of the leading causes of mortality and morbidity worldwide [1]. Recent evidence revealed that over 600,000 deaths and about two million new cases are projected to occur in 2022 in the United States of America [1]. Meanwhile, prostate cancer has long been known as the most common non-cutaneous malignancy diagnosed worldwide in the male population [2]. According to recent statistics, it is the second leading cause of cancer-related death after lung cancer [2]. During the last decade, the proportion of prostate cancer diagnosed at a distant stage has increased from 3.9% to 8.2% [1]. This complex disease is assumed to be prevalent in areas with a high human development index [3][4]. Advancing age, race, lack of physical activity, family history, a fat-enriched diet, obesity, sexual activity, smoking, alcoholism, and occupation are the main risk factors for prostate cancer [5]. Also, a variety of genetic and epigenetic alterations, such as androgen receptor hyperactivation by amplification and/or mutation, amplification of the *MYC* oncogene, *PD-L1*expression, deletion and/or mutation of *PTEN* and *TP53*, long noncoding and microRNA dysregulation, fusions of TMPRSS2 with ETS family genes, could be resulted in the incidence, development, and progression of prostate cancer [6][7].

Fortunately, 99% overall survival for almost ten years is estimated for prostate cancer patients with localized disease, which is significantly dependent on early detection [8]. Digital rectal examination, prostate-specific antigen, genetic and epigenetic tests (including breast cancer genes 1 and 2, checkpoint kinase 2, mutL homolog 1, BRCA1-interacting protein C-terminal helicase 1, postmeiotic segregation increased 2, mutS homologs 2 and 6, homeobox B13, nibrin, and ataxia telangiectasia mutated), as well as prostate biopsy are listed as current diagnostic approaches in the clinic [9]. However, the insurance coverage for testing, bridging the gap between clinical and diagnostic disciplines concerning novel biomarkers and nanotechnology platforms, and the low sensitivity and specificity are documented as current limitations that could lead to early detection failure [10].

Due to the complex aspects of prostate cancer, the recommended therapeutic strategies represent unique complexities [11]. Interestingly, unlike other solid tumors, chemotherapy is not considered the first line of treatment for prostate cancer, but the choice of the best treatment option strikingly depends on the level of tumor progression [11][12]. For localized prostate cancer, defined as no identifiable regional lymph nodes or distant metastases, expectant management, surgery, and radiotherapy continue to be standard curative treatments [13]. Expectant management is considered the monitoring for prostate cancer progression without undergoing any definitive therapy, includes watchful waiting that involves treating symptoms with alleviative intent, and active surveillance that consists of a series of prostate biopsies, physical examinations, prostate-specific antigen (PSA) testing, or its combination to monitor tumor progression and choice the proper decision in different conditions [14]. In addition, surgery and radiation are considered effective treatments for patients with more severe diseases, such as those with a PSA level greater than 10 ng/mL and patients whose digital rectal examination revealed palpable nodules [15][16]. However, the quality of life of survivors is negatively affected by significant adversities such as sexual dysfunction and urinary symptoms [17].

## The Trend of Chemotherapeutic Development for Treatment Prostate Cancer

For patients with more advanced metastatic prostate cancer, many efforts were made during the 1970s and 1990s, and various chemotherapeutics were designed and tested in phase II settings [18]. However, none of these chemotherapy agents received final approval, except estramustine [18]. Estramustine was comprised of two moieties, an estrogenic chemical and an alkylating agent, which these two parts split upon cellular uptake [19]. Previous;y, assumed that the alkylating content affects the DNA of the neoplastic cells, and the estrogenic component reverses the effects of androgens. While in later, it was clarified that the antitumoral properties of estramustine against advanced prostate cancer were not based on any of these assumptions; rather, its antineoplastic effects were due to the binding and depolymerization of microtubules [19][20]. Currently, other chemotherapeutics, such as anthracenedione mitoxantrone, known as novantrone, have been approved [21]. However, disease progression, treatment failures, lack of improvement in the quality of life of survivors, and cardio-, haemato-, and hepatotoxicity were mentioned as the main adverse effects of mitoxantrone [21][22]. While taxanes were preferred due to their more desired efficacy and improved quality of life for survivors [23]. The different types of taxanes, including docetaxel (Taxotere), paclitaxel (Taxol), and cabazitaxel (Jevtana) were derived from the Pacific Yew tree and similar to estramustine were able to stabilize microtubules that are followed by the mitotic block, activation of proapoptotic Bcl-2, and androgen-dependent signaling all of which leading to cancer cell apoptosis [23]. Despite the acceptable impact of taxanes on the survivors' quality of life, the resistance mechanisms in metastatic castration-resistant prostate cancer led to the limited efficacy of taxanes and subsequent efforts to propose alternative chemotherapeutics [24]. Multidrug resistance related to the expression of ATP-binding cassette transporter family on cancer cells, the epithelial-mesenchymal transition, androgen receptor splice variant expression (e.g., AR-V7), and tubulin alterations (e.g., overexpression of the β-tubulin III isoform) are important resistance mechanisms for taxans [24]. Non-taxane chemotherapeutics such as cyclophosphamide and carboplatin revealed no proven overall survival benefit [23][25]. Meanwhile, the chemotherapeutics that benefit from androgen receptor properties were developed.

## Androgen Deprivation and Anti-Androgen Chemotherapeutics

The formation of prostatic intraepithelial neoplasia is the initiation step of prostate cancer progression, followed by high-grade prostatic intraepithelial neoplasia, adenocarcinoma, and metastasis [26]. The growth and function of the prostate are heavily reliant on androgens. Hence, androgen deprivation is assumed to be a therapeutical strategy due to the beneficial consequences such as prostate involution, reduction in size, and induction of cellular senescence in prostate cancer cells [26]. Prostate tissue is severely dependent on androgens such as testosterone and dihydrotestosterone (DHT) in both physiological and pathological conditions; hence, androgen deprivation has long been considered a key mechanism for preventing the growth and progression of castration-resistant prostate cancer [26]. For the first time, John Hunter introduced the association between prostatic disease and androgen ablation in 1786s [27]. However, in 1941, Huggins *et al*. determined that androgen deprivation is an effective strategy for treating metastatic castration-resistant prostate cancer [28].

Although radical prostatectomy and/or radiation therapy are considered the standard treatment for patients with localized prostate cancer, up to one-third of patients must deal with a biochemical recurrence [29]. In this stage, androgen deprivation therapies with and without chemotherapy agents (such as taxanes) are administered, depending on metastasis status [29]. The orchiectomy and the administration of the gonadotrophin-releasing hormone inhibitor, such as goserelin, degarelix, and leuprolide, are considered the main strategies that cause androgen deprivation; in other words, dropping the levels of testosterone [30][31]. Leuprolide and goserelin are approved luteinizing hormone-releasing hormone (LHRH) agonists, whereas degarelix is an approved LHRH antagonist [32]. All these three chemotherapeutics reduce the levels of luteinizing hormone biosynthesis, followed by decreased levels of testosterone produced by gonads [33]. Despite the suitable efficacy of these pharmaceutics, which caused their consideration as chemotherapeutical alternatives to surgical castration, significant limitations, including the continuous production of androgens by adrenal and the synthesis of testosterone by the prostate cancer cell itself lead to investigates of other therapies (e.g., blocking androgen receptor) [34].

Evidently, androgens induce both desired or catastrophic effects on target cells through the androgen receptor [34]. The androgen receptor consists of four distinct domains, including the C-terminal ligand binding domain (LBD), the hinger region that is required for N- and C-terminal interaction, the DNA binding domain (DBD), and the N-terminal domain [34]. The inactivated form of the androgen receptor is located in the cytoplasm of target cells, binding to different chaperone proteins [34]. The attachment of androgens to the LBD causes the release of bonded chaperons that lead to the homodimerization of the receptor and its translocation to the nucleus [34]. In the nucleus, the androgen receptor behaves like a transcriptional factor and modifies the expression of androgen-responsive genes, such as the PSA [35]. Interestingly, the degradation of the androgen receptor, the prevention of androgen receptor nuclear translocation, the blockade of androgen receptor N- to C-terminal interaction, the sequestration of DHT ligands that activate androgen receptor, the disruption of androgen receptor co-activator interaction, and the inhibition of downstream signalings are documented as androgen receptor aspects that could exert therapeutical benefits [36]. The first attempts led to the development of the first generation of anti-androgens for metastatic castration-resistant prostate cancer.

## First-Generation Anti-Androgens

Flutamide, nilutamide, and bicalutamide are the main first-generation anti-androgens that are United States Food and Drug Administration (U.S FDA) approved and clinically prescribed for patients with advanced prostate cancer [37].

Flutamide was first discovered in 1967 when its formulation was used as an antibiotic. However, its anti-androgenic properties were later found in 1971 by The Schering-Plough Corporation of Germany [37]. Upon approval from the U.S FDA in 1989, flutamide became the first non-steroidal anti-androgen chemotherapy [37]. The FDA is recommended flutamide for patients with stage B2-C and stage D2 metastatic prostate cancer [37]. Nilutamide, with a similar structure to flutamide, was approved for clinical use in the U.S in 1996 [38]. This oral agent could block the binding of androgens to its receptors, thereby representing its therapeutic properties against prostate cancer, such as reducing pain and preventing disease progression [38]. Nilutamide is mainly administrated for patients with metastatic prostate cancer who underwent orchiectomy or received other anti-androgens like LHRH agonists [38]. Accordingly, bicalutamide is another non-steroidal anti-androgen agent with a similar structure to flutamide and nilutamide [38]. This orally available chemotherapeutic was approved in the U.S one year earlier than nilutamide and exerts favored properties like two other first-generation anti-androgens; hence it can block androgen-stimulating effects in androgen-sensitive tissues such as the prostate, testis, hypothalamus, breast, and skin [38]. Also, the combination of bicalutamide with other anti-androgens, such as leuprolide or goserelin, is recommended for patients with advanced prostate cancer [38].

## Adversities of First-Generation Anti-androgens on the Cardiovascular System

Despite the effectiveness during the early decade of approval and administration of the first-generation anti-androgens, their application was limited due to androgen receptor resistance and unexpected adverse effects on normal cells that led to decreased bone mineral density, sexual dysfunction, hot flashes, metabolic dysregulation, as well as cardiac morbidity and congenital dysfunction [39].

Although androgen deprivation therapy initially demonstrates high response rates, the majority of prostate cancer cases develop inevitable castration-resistance, mostly in one year after such treatment [40]. To understand the mechanism of androgen receptor resistance, it is reasonable first to describe the mechanism of action of this receptor. Importantly, the androgen receptor plays a pivotal role during the androgen-dependent progression of prostate cancer cells [41]. Upon the entrance of testosterone to the cancer cells, the 5a reductase enzyme converts this androgen to its active metabolite known as DHT [41]. The binding of DHT to the androgen receptor located in the cytoplasm leads to the translocation of the receptor to the nucleus, its attachment to androgen-response elements, and the activation of genes related to cell growth [41][42]. However, in the absence of androgen, prostate cancer cells could develop a variety of cellular pathways to survive, called androgen-independent progression [43]. In the hypersensitive pathway, for example, the prostate cancer cell produces more androgen receptors that could be activated despite reduced active metabolite levels [43]. More androgen receptors could be achieved by the ability of prostate cancer cells to develop androgen receptor gene amplification via mutations or similar mechanisms. Creating a promiscuous receptor is another mechanism by which prostate cancer cells alter the specificity of the androgen receptor by mutations that lead to the activation of the androgen receptor by nonandrogenic steroids normally present in the cell or circulation [43]. The nonandrogenic steroids-mediated activation of the androgen receptor, which requires the phosphorylation of the receptor, can be done through two separate pathways, including the bypass and the outlaw pathways [43]. In the bypass pathway, the survival of prostate cancer cells occurs independent of the androgen receptor activation, for example, the upregulation of Bcl2 proapoptotic protein by androgen-independent prostate cancer cells that inhibits their apoptosis and thereby increase their survival [43][44]. In the second pathway, the irregulated cytokines and growth factors directly phosphorylate the receptor and thus activate it. In addition, the secretion of specific neuropeptides by neuroendocrine cells could support and induce the growth and progression of androgen-independent prostate cancer cells. More likely, the presence of prostate cancer stem cells within the prostate tumor could support the growth of androgen-independent cancer cells [44]. Unfortunately, these masterpiece cellular mechanisms led to the lack of dependence on androgens and eventually caused prostate cancer to become unresponsive to first-generation anti-androgens [44].

In addition to the lack of response to the first-generation anti-androgens, cardiotoxicity and cardio morbidity after treatment are considered to be these chemotherapeutics' most important limiting factors. It is evidenced that heart failure (HF), QTc interval prolongation, and atrial fibrillation could occur in patients with advanced prostate cancer who received first-generation anti-androgens resulting from dysregulated lipid profile, reduced insulin sensitivity, inflammatory reactions, induction of prothrombotic state, the formation of atherogenic plaque, and plaque destabilization [45]. The initial evidence indicated that the first-generation anti-androgens did not have a detrimental consequence on the lipid profile because the performed studies until 2003 indicated the similarity of the recipient groups with the placebo group [46]. Importantly, bicalutamide could cause HF in elderly patients with prostate cancer via the induction of apoptosis revealed by altered ET-1, Bcl2, and cyclin-A [47]. In addition, the recent scientific statement from the American Heart Association declared undesired effects on the cardiovascular system after receiving all three types of first-generation anti-androgens, including bicalutamide (peripheral edema, hypertension, angina, myocardial ischemia, HF, cardiac arrest, and vasodepressor syncope), flutamide (edema and hypertension), and nilutamide (QT prolongation, hypertension, HF, edema, and vasodepressor syncope) [48].

Furthermore, androgen deprivation therapy for more than one year has been associated with a 20% higher risk of cardiovascular morbidity [49]. The deleterious effects induced after testosterone deprivation can be related to the cardioprotective properties of testosterone, such as maintaining lipid profile and prevention of inflammation through its androgen receptor and modification of various signaling pathways [50]. To compensate for the lack of efficiency and unfavored adverse effects, the researchers studied the design of novel chemotherapeutics as well as the coadministration with herbal medicinal compounds that provide antitumor properties and reduce side effects as two main desired strategies [51]. It was at this point that the second-generation anti-androgens emerged.

## Second-Generation Anti-androgens

The second-generation anti-androgens, also known as next-generation anti-androgens, were presented to the clinic to overcome the first-generation's inefficiency in dealing with the resistance of the androgen receptor to these compounds and ensuring the safety of this antitumor family. Currently, four second-generation anti-androgens, including abiraterone acetate, enzalutamide, apalutamide, and darolutamide have been approved for the treatment of advanced prostate cancer. Indeed, the U.S FDA and the National Comprehensive Cancer Network guidelines have approved and classified all of these compounds as first-line treatment options for metastatic castration-sensitive prostate cancer and have also recommended them for nonmetastatic castration-resistant prostate cancer [52].

### Abiraterone Acetate

Abiraterone is considered the predecessor to abiraterone acetate and was discovered based on its ability to inhibit cytochrome P450 17α-hydroxylase/17,20-lyase (CYP17) enzyme [53]. The background of the discovery of this anti-androgen refers to the ability of a well-known antifungal, ketoconazole, which was an inhibitor of CYP17 and was suggested to be capable of preventing the growth of prostate cancer; however, a low potency, the induction of adrenal insufficiency, and its short half-life were determined as its major limitations [53]. As a CYP17 inhibitor, abiraterone acetate can decrease the levels of testosterone production in the whole body. Many clinical trials were conducted to evaluate the efficacy of abiraterone acetate in the treatment of prostate cancer; however, the non-specific inhibition of other members of the CYP family led to the high toxicity of this compound [54]. The synthesis of abiraterone with altered structure was considered the main strategy to overcome its adverse effects resulting in several different abiraterone compounds with steroidal scaffolds replaced with non-steroidal cores [54]. The abiraterone acetate was the most well-known altered compound with a non-steroidal scaffold, low toxicity, and higher selectivity to target CYP17 [54]. As a result, abiraterone acetate was named the first next-generation anti-androgen approved by FDA in April 2011 [55]. It is considered the only anti-androgen that inhibits androgen biosynthesis by targeting CYP17 instead of inhibiting the androgen receptor, like canonical anti-androgens [55].

Although the different mechanism of action of abiraterone acetate, the inhibition of the CYP17 enzyme activity and consequently a remarkable reduction in the production of androgens, led to a logical inexpectation for the lack of androgen receptor resistance, other mutations have made prostate cancer cells capable of resistant to this chemotherapy [55][56]. For example, overexpression or genomic alterations in the CYP17A1 enzyme are demonstrated to contribute to abiraterone acetate resistance [56]. Furthermore, it is revealed that the HSD3B1 (1245C) mutation resulted in abiraterone acetate resistance progression to castration-resistant prostate cancer [57]. Despite initial efficacy in the treatment of prostate cancer patients and even in the successful suppression of testosterone production, the androgen receptor re-activation drives prostate cancer to lethal castration-resistant prostate cancer phenotype in all treated patients [58].

### Enzalutamide

Enzalutamide was first developed by the isolation of a mutagenic screen of the non-steroidal agonist RU59063 [58]. By the examination of different compounds as antagonists of the androgen receptor and optimization of stability and bioavailability, enzalutamide was discovered to represent 5- to 8-fold greater affinity to the androgen receptor-slightly less affinity than androgen receptor ligand DHT-than other studied compounds [59]. Furthermore, it was established that this newly discovered anti-androgen could interrupt the nuclear translocation of the androgen receptor and inhibit androgen receptor binding to DNA and co-activator recruitment [59]. In contrast to first-generation anti-androgens, enzalutamide did not exhibit agonist androgen receptor activities [59]. Enzalutamide was approved in 2012 by the U.S FDA for the treatment of patients with metastatic castration-resistant prostate cancer [55].

Resistance to enzalutamide in prostate cancer cells could be classified into two distinct categories: androgen receptor-dependent and androgen receptor-independent mechanisms [59]. A variety of genetic alterations in the androgen receptor, such as gene amplification, translocation of driver genes, and mutations that are followed by androgen receptor splice variants and point mutations, could considered as the main receptor-dependent mechanisms of enzalutamide [60]. Notably, it is evidenced that a vast number, as large as half of the patients pretreated with enzalutamide, exhibited androgen receptors, which was accompanied by the response to treatment in only 13% of them [60]. Furthermore, up to 30% of mutations in the androgen receptor occur in circulating tumor cells and circulating DNA of patients with castration-resistant prostate cancer leading to resistance to enzalutamide [61]. Some androgen receptor mutations make enzalutamide a receptor agonist instead of its antagonist properties [61]. In addition, different mutations in LBD, repeated sequences, and evidenced polymorphisms result in a lower affinity of enzalutamide to the androgen receptor and failure of anti-androgen therapy [62]. Along with that, androgen receptor-independent mechanisms include induced alterations in different signaling pathways such as PI3K/AKT, glucocorticoid, NF-ҡB, FOXO, Wnt, cytokines, autophagy, epithelial-mesenchymal transition, neuroendocrine differentiation, immune resistance, and TMPRSS2-ERG fusion signalings all of which leading to lower efficacy of enzalutamide [63][64].

### Apalutamide

The third next-generation anti-androgen is named apalutamide, an androgen receptor antagonist whose development is based on the knowledge gained in the manufacture of enzalutamide. The mechanism of action of apalutamide refers to its high affinity to LBD of androgen receptor leading to the inhibition of its nuclear translocation and DNA binding [65]. The generation of this synthetic compound based on the structure-activity relationship-guided chemistry endure its permanent antagonistic activity when the androgen receptor is expressed. Indeed, apalutamide shares similar properties with bicalutamide as these two anti-androgens binds to a site on the androgen receptor; however, the affinity of apalutamide is estimated to be 7- to 10-fold higher than bicalutamide [65]. Another desired merit of apalutamide in comparison with bicalutamide is its remained affinity of this compound to the androgen receptor, even in the overexpression setting of the receptor [65]. In addition, compared to enzalutamide, apalutamide represents higher efficacy, less crossing of the blood-brain barrier (BBB), and higher concentrations in the tumor than plasma, all of which suggest its enhanced antitumor activity along with less adverse effects on the central nervous system (CNS) [65]. Upon underwent through various phases of clinical trials, finally, the third next-generation anti-androgen received FDA approval in February 2018 and was recommended as the first line of treatment for patients with metastatic castration-resistant prostate cancer [66].

According to the recent approval of apalutamide, few studies are available to determine drug resistance. On the contrary, Smith *et al*. revealed that treatment with apalutamide in nonmetastatic castration-resistant prostate cancer patients was not followed by a significant increment in the frequency of the androgen receptor anomalies that are common in the androgen receptor-signaling targeted therapy-resistant metastatic castration-resistant prostate cancer [67]. So, it represents apalutamide potential as a circumvent mechanism of resistance to common androgen deprivation therapies [67]. In addition, despite in vivo reports mentioning that mutations in the androgen receptor, such as F877L and T878A mutations, cause partial conversion of apalutamide from receptor antagonist to its agonist, clinical trial studies, whether phase I or II, have not found these mutations to lead to the acquisition of drug resistance [68].

### Darolutamide

With approval in August 2022, darolutamide became the latest member of second-generation anti-androgens recommended by the FDA for the treatment of advanced prostate cancer [69]. This androgen receptor antagonist or its active metabolite, known as ORM-15341, inhibits the receptor nuclear translocation [70]. Interestingly, darolutamide not only demonstrates a higher affinity to the androgen receptor and more potency to inhibit the receptor than apalutamide and enzalutamide, along with low penetrance of the BBB but also has the ability to inhibit the wild-type androgen receptor and clinically relevant androgen receptor mutations that are resistant to enzalutamide and apalutamide; hence, exhibiting a unique therapeutic potency against advanced prostate cancer resistant to other standard chemotherapeutics [71].

The absence of evidence of drug resistance until now, along with less toxicity in the CNS and high binding affinity even in androgen receptor mutant species, are among the merits of darolutamide compared to other next-generation anti-androgens [72]. However, due to its low bioavailability, the compulsion to take darolutamide with food is considered its just disadvantage, particularly compared to enzalutamide and apalutamide [52][72].

## Cardiotoxicity of Second-Generation Anti-Androgens

As HF and cardiovascular disorders have been one of the most important consequences of treating prostate cancer patients with first-generation anti-androgens, and as a result limiting their recommendation as the first option of physicians, a similar concern about the next-generation anti-androgens has emerged in researchers that have been the basis for several studies in this field.

An open-label, single-arm phase I study revealed that abiraterone acetate and its active metabolite, abiraterone, cause no adverse effects on QT/QTc interval [73]. Hence, the administration of this firstly approved member of second-generation anti-androgens is not followed by human ventricular repolarization suggesting its significant cardio safety [73]. However, atrial fibrillation, frequent premature ventricular contractions, interrupted QTc, and refractory hypokalaemia was reported in an elderly patient with metastatic castration-resistant prostate cancer who received abiraterone [74]. A most recent cell-free DNA sequencing and lipid profile analysis on 106 patients with metastatic castration-resistant prostate cancer who received different chemotherapeutics (e.g., abiraterone acetate and enzalutamide) showed aberrations in the androgen receptor, *TP53*, RB1, PI3K, and aggressive-variant prostate cancer along with lipid abnormalities [75] are well-known pieces of evidence to be risk factors for cardiovascular disorders [76]. Furthermore, a case report showed that the administration of abiraterone acetate to a 70-year-old male resulted in cardiovascular dysfunction and syncope [77]. In a cohort of 174 patients with metastatic castration-resistant prostate cancer treated with abiraterone approximately 25% experienced the development or deterioration of cardiovascular and metabolic disorders [78]. Moreover, a pharmacovigilance study demonstrated that HF and atrial tachyarrhythmia are the main adverse effects of abiraterone therapy [79]. Cardiac dysfunction and HF four weeks after administration of abiraterone is reported in a patient with castration‑resistant prostate cancer [80].

The primary distribution of enzalutamide in the cardiocytes could be considered a potential risk factor for possible cardiovascular consequences [81]. A retrospective study revealed that anti-androgen therapy (such as abiraterone and enzalutamide) in patients with metastatic castration-resistant prostate cancer was associated with a higher risk of metabolic syndrome, cardiovascular disorders, and neurological dysfunction [82]. Similarly, an increased risk of acute coronary syndrome, HF, and ischemic stroke are reported in castration-resistant prostate cancer patients who received abiraterone or enzalutamide [83]. It appears that cardiovascular adversities caused by abiraterone and/or enzalutamide are more expected in treated elder patients [84]. Also, a pharmacovigilance study showed that cardiovascular toxicities are attributed to abiraterone but not enzalutamide [85]. In addition, short-term enzalutamide therapy is suggested to be heart-healthy [86]. However, in elderly patients with advanced prostate cancer and/or hypogonadism, six months of enzalutamide therapy resulted in the variation of cardio-electrophysiological balance, including prolongation of QTc, QTd, maxTpe, mean Tpe/QT, maxTpe/QT, and Tped [87]. Similarly, ventricular repolarization is reported as a possible adverse effect of enzalutamide therapy in male patients with hypogonadism [88]. Furthermore, enzalutamide could cause toxic myocarditis by affecting a co-regulator that interacts with and hyperactivates the rs5522-mutated mineralocorticoid receptor [89]. A randomized, double-blind, phase II study documented high grades of cardiac adverse events such as myocardial infarction, congestive HF, and atrial fibrillation upon treatment with enzalutamide [90].

Due to the recent approval of two other members of second-generation anti-androgens, particularly darolutamide, information regarding these chemotherapeutics-induced cardiotoxicities is not well reported. An open-label phase Ib study in patients with castration-resistant prostate cancer demonstrated that apalutamide therapy did not cause QTc prolongation and ventricular repolarization [91]. A randomized, placebo-controlled, phase III study on 1052 patients with metastatic castration-sensitive prostate cancer that evaluated health-related quality of life after apalutamide treatment reported no occurrence of any cardio-related adversities [92]. However, ischaemic heart disease and HF are reported in almost 2% of patients who received apalutamide [93]. In addition, in the Japanese population who underwent apalutamide therapy, a few (3.6%) patients with metastatic castration-sensitive prostate cancer experienced ischemic heart disease (IHD) and angina pectoris [94]. Similarly, in patients who received darolutamide, HF was observed in 1.9% of patients [95]. Interestingly, in Japanese nonmetastatic castration‑resistant prostate cancer patients, no evidence of any cardiotoxicity related to darolutamide therapy was reported [96]. Furthermore, an adequate number of patients with grade 3 or 4 of cardiac adversities, including HF (0.4%), coronary artery disorder (2%), cardiac arrhythmia (1.8%), and hypertension (3.5%) were reported after darolutamide therapy [97].

## Conclusion

The reviewed studies revealed that mutation and other genetic anomalies resulted in prostate cancer cells' resistance to two firstly approved members of next-generation anti-androgens, including abiraterone acetate and enzalutamide. Furthermore, cardiotoxicity, such as HF, ventricular repolarization, hypertension, myocarditis, atrial fibrillation, and IHD, is frequent in patients who underwent abiraterone acetate and/or enzalutamide therapy. However, the other two members, apalutamide and darolutamide, which have recently been approved for patients with advanced prostate cancer, have no concerns about genetic alterations in the androgen receptor. As a result, the lack of response to treatment by prostate cancer cells has not been reported. Moreover, cardiotoxicity has rarely been observed after apalutamide and/or darolutamide therapy. Therefore, it seems that the administration of abiraterone acetate and enzalutamide, especially in high-risk individuals such as the elderly and those with a history of cardiovascular disease, diabetes, hypertension, metabolic syndrome, and impaired lipid profile, should be accompanied by more medical care as well as consultation of other related specialists. Even though the studies indicate that apalutamide and darolutamide are safe and effective, the insufficiency of the information and the necessity for further studies are clearly evident.

## Conflict of Interest

The authors declared that have no conflict of interest.
